# No difference in clinical outcome, pain, and range of motion between fixed and mobile bearing Attune total knee arthroplasty: a prospective single-center trial

**DOI:** 10.1186/s12891-022-05382-x

**Published:** 2022-05-02

**Authors:** Paul Ruckenstuhl, Fabio Revelant, Georg Hauer, Gerwin A. Bernhardt, Lukas Leitner, Gerald Gruber, Andreas Leithner, Patrick Sadoghi

**Affiliations:** Department of Orthopedics and Trauma, Medical University of Graz, Auenbruggerplatz 5, A-8036 Graz, Austria

**Keywords:** Total knee arthroplasty, Fixed bearing, Mobile bearing

## Abstract

**Background:**

Despite numerous scientific investigations, the tribological advantages of mobile bearing inserts have not been sustainably confirmed or refuted for modern knee prostheses in clinical studies. The purpose of this study was to compare fixed and mobile bearing inserts in order to draw conclusions regarding clinical benefits.

**Methods:**

The present prospective single center cohort study of 2 non-randomized stratified groups consisted of 67 patients. All included patients received cemented total knee arthroplasty (Attune®) due to osteoarthritis. 34 patients were treated with a mobile and 33 patients with a fixed insert. The WOMAC score and the Visual Analogue Scale was used for the subjective assessment of success, while the Knee-Society-Score was used considering the Range of Motion for the objective assessment. The subjective and the clinical scores showed improvements for both compared groups postoperatively at 2 years of minimum follow-up.

**Results:**

The overall postoperative results of the WOMAC score, the Knee-Society-Score and the Visual Analogue Scale presented no statistically difference between the compared groups (*p* > 0,05). The postoperative ROM showed a superior improvement of 13.2° ± 18.4° in the mobile-bearing group versus 4.9° ± 18.4° (*p* = 0.017) in the fixed-bearing group. The flexion of the knee joint was 114° ± 10.1° for the mobile-bearings and 109.2° ± 7.2° for fixed bearings (*p* = 0.012).

**Conclusion:**

According to the findings, both inserts showed overall promising postoperative results, in terms of objective as well as subjective parameters, without clinically relevant significant differences, except for ROM, which was superior in the mobile bearing group.

The present clinical trial has been registered at the ISRCTN registry with the reverence number ISRCTN15117998 on 04/04/2022.

**Supplementary Information:**

The online version contains supplementary material available at 10.1186/s12891-022-05382-x.

## Introduction

In the past, the use of mobile (MB) and fixed bearing (FB) inserts were discussed controversially in the literature [1–6]. MB were designed to allow rotation of the insert around the longitudinal axis between the insert and the tibial component [7]. It is postulated in the literature that lower shear forces due to movement of the insert relative to the tibial tray may decrease the rate of insert wear and implant loosening as well as the rate of osteolysis [7–9]. Furthermore, superior postoperative ROM and joint function has been published for MB inserts due to the accommodation of rotational mismatch [10–12]. These postulated advantages made the use of MB inserts popular for many surgeons. On the other hand, concerns regarding the risk of insert dislocation, soft tissue impingement and postoperative instability are associated with MB inserts [9, 13–15]. To prevent insert dislocations an exact balancing in flexion and extension is recommended [16]. Therefore, the use of MB insert is considered as technically more challenging and associated with a prolonged learning curve [9, 13, 17]. It has been published that the additional articulating surface on the underside of MB inserts could encourage wear [5, 9].

Several studies have analyzed the clinical differences between MB and fixed bearing inserts, mostly without any significant results in favor or against one treatment option for cemented Total Knee Arthroplasty (TKA) [17–20]. Simultaneously, total knee prostheses have substantially improved, especially in terms of the quality of polyethylene and fixation methods [21]. Recent long term randomized controlled trials with large cohorts and literature reviews reported of no differences in durability, function, range of movement and migration [5, 8, 12].

Modern total knee prostheses, such as the presented implant type (Attune®) provide a transition from stability and rotational freedom. This is the first study to analyze subjective and objective measurements between FB and MB inserts of this well-established TKA system.

The study hypothesizes that the use of fixed or mobile bearing inserts do not differ significantly in the presented total knee system.

## Material and methods

**Fig. 1:**
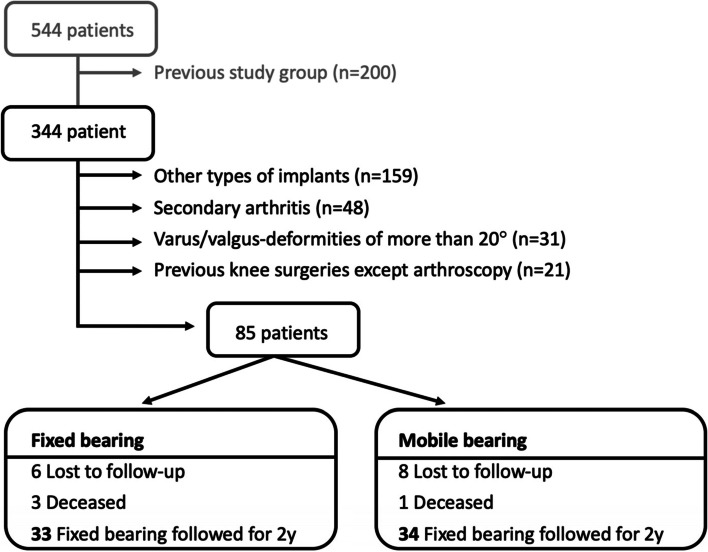
Flow chart on inclusion of patients

Between January 2015 and December 2016, a total of 544 primary total knee arthroplasties were implanted at one single orthopedic center. During this period another prospective level II study was conducted selecting patients of this time interval (*n* = 200). The remaining 344 patients were eligible for the presented study (Fig. 1**)**. All patients either received a fixed or mobile bearing insert. The decision whether a patient received a MB of FB insert was made prior to the surgery based in a non-randomized setting. During the operation the selection of insert type was not changed in any case. Patients with a secondary arthritis, previous knee surgeries except arthroscopies, and varus/valgus-deformities of more than 20° were excluded. Moreover, we only included patients treated with one well-proved and worldwide used type of implant (Attune®, DePuy-Synthes, Warsaw, Indiana). All implants were tibial and femoral fixed with cement.

### Study population

Finally, the present prospective cohort of 2 non-randomized stratified groups consisted of 67 patients, 26 males and 41 females respectively, that met the inclusion criteria. All included patients received either a MB or FB insert of the same designed posterior cruciate ligament preserving type of knee prosthesis.

Thirty-four patients received MB inserts and 33 FB inserts **(**Fig. 1**)**. The MB group consisted of 10 males and 23 females, whereas the FB group included 16 males and 18 females. Overall the mean age at the time of operation was 66.6 (48–79) years. Patients treated with a MB insert were 65.7 (48–79) years old and with FB inserts 67.5 (50–79) years. The mean follow-up time from surgery to the last examination was 46 (25–60) months. The mean follow-up was 39 months for the fixed-bearing and 53 months for the mobile bearing group. Overall, the mean BMI for all patients was 30.6 (21–44) and did not differ significantly between the 2 study groups **(**Table 1**).**

**Table 1:** Patient demographics and baseline characteristics

Demographics	Fixed-bearing (***n*** = 33)	Mobile-bearing (***n*** = 34)	***p***-value
Sex (M/W) (n/%)	10 (30%)/23(70%)	16(47%)/18(53%)	0.159
Age (years), mean (range)	65.7 (54–78)	67.5 (51–79)	0.346
BMI (kg/m^2^), mean (range)	31.8 (21–44)	29.3 (23–38)	0.052

All operations were performed by two board certified orthopedic surgeons (GG and PS) with an experience of more than 10 years including at least 50 cases per year. Surgery was performed by using a standard medial parapatellar approach and the resection was performed primarily tibial. All components were cemented and during cementation a tourniquet was used. The articular surface of the patella was not replaced in any case. Postoperatively, all patients underwent a standardized physiotherapeutic regime including a stationary rehabilitation program. Radiographs were performed preoperatively and postoperatively during the hospital stay as well as 6 weeks, 6 months postoperatively, and at the time of last follow-up. All clinical examinations were performed preoperatively during the admission and postoperatively at the outpatient department.

Indication for surgery was based on x-ray images (Kellgren-Lawrence-Score III/IV) and clinical examination during a visit in the knee specialized outpatient department of clinic. Postoperative, all included patients completed a standardized rehabilitation protocol. Full weight bearing with 2 crutches was allowed immediately after surgery and a postoperative therapy with continuous passive motion (CPM) started on the first day after surgery.

### Clinical measurement

Pre- and postoperatively the Knee Society Score (KSS) was assessed to evaluate pain and knee function [22]. Range of Motion (ROM) as measured with a goniometer. The ROM was defined as the degrees of knee flexion minus the amount of extension deficit or plus the amount of hyperextension. Moreover, the visual analogue scale (VAS) was applied as pain level measurement.

### Patient related outcome measurement (PROM)

The WOMAC (Western Ontario and McMaster Universities Osteoarthritis Index) score was implemented as a patient reported outcome measurement (PROM) [23]. This score includes 24 questions and allows predictions regarding pain, stiffness, and physical function of the knee joint. The score has been especially developed for patients suffering from osteoarthritis.

Radiographs were made preoperatively and postoperatively before patients left the hospital, six weeks postoperatively as well as during the follow-up examination. All methods were carried out in accordance with relevant guidelines and regulations.

Complications were analyzed according to Goslings and Gouma [24].

### Statistical analysis

All data were analyzed by SPSS Version 22.0 (IBM Corporation, New York, USA). Descriptive statistics for continuous variables were reported as the mean and standard deviation (SD). Categorical variables were reported as count and proportions. For comparisons of categorical variables, the chi-square exact test was used. Data were tested for normality using Kolmogorov-Smirnov test. Differences between pre-operative and post-operative data were observed with Mann-Whitney U test and Wilcoxon signed-rank test. An a priori power analysis initially was performed and a *p*-value less than 0.05 was defined as statistically significant.

## Results

### Clinical results

Postoperatively, ROM was statistically significantly superior in the group of patients treated with MB inserts with a mean ROM of 114° (80–130) compared to and 109.2° (100–125) for the FB group (*p* = 0.012). Furthermore, the increase of movement during the follow-up period was significantly superior for MB inserts (*p* = 0.017). Compared to the preoperative ROM, patients with MBs showed an improvement of 13.2° ± 18.4 and with FBs of 4.9° ± 14.8 **(**Table 2**)**.

**Table 2:** Comparison between Attune mobile-bearing (MB) and Attune fixed-bearing (FB) before surgery and after a two-year follow-up examination. Results presented as mean and standard deviation. The *p*-value was set as *p* > 0.05

	Mobile-bearing (***n*** = 34)	Fixed-bearing (***n*** = 33)	*p*-value
**ROM (°) (mean ± SD)**			
Pre-operative	100.7 ± 14.5	104.4 ± 18.3	*p* = 0.081
Post-operative	114.0 ± 10.1	109.2 ± 7.2	***p*** **= 0.012**
Change in ROM	13.3 ± 18.4	4.9 ± 18.4	***p*** **= 0.017**
**KSS pain (mean ± SD)**			
Pre-operative	56.9 ± 9.3	58.7 ± 15.4	*p* = 0.099
Post-operative	92.6 ± 10.3	94.2 ± 5.8	*p* = 0.645
Change in KSS pain	35.7 ± 13.5	35.5 ± 16.4	*p* = 0.386
**KSS function (mean ± SD)**			
Pre-operative	47.2 ± 13.3	48.5 ± 13.7	*p* = 0.871
Post-operative	83.1 ± 20.5	86.5 ± 16.8	*p* = 0.601
Change in KSS function	35.9 ± 24.4	38.0 ± 18.6	p = 0.790
**WOMAC (mean ± SD)**			
Pre-operative	57.7 ± 10.9	51.0 ± 13.8	***p*** **= 0.033**
Post-operative	86.1 ± 14.9	90.3 ± 9.5	*p* = 0.386
Change in WOMAC	28.3 ± 17.1	39.3 ± 14.5	***p*** **= 0.018**
**VAS pain (mean ± SD)**			
Pre-operative	6.6 ± 2.0	7.2 ± 1.8	*p* = 0.161
Post-operative	1.7 ± 1.4	1.6 ± 1.0	*p* = 0.973
Change in VAS	4.8 ± 2.5	5.6 ± 2.0	*p* = 0.224

*SD* Standard deviation, *MB* Mobile-bearing, *FB* fixed bearing, *KSS* knee society score, *WOMAC* Western Ontario and McMaster Universities Osteoarthritis Index, *ROM* range of motion, *VAS* Visual Analogue Scale

No significant difference was observed between pre- and postoperative results regarding the KSS function (*p* = 0.790) and KSS pain (*p* = 0.386). Patients treated with FB inserts reached higher postoperative results. Both groups presented almost the same level of increase for KSS pain during the follow up period. Regarding KSS function results, patients treated with FB inserts increased stronger during the follow-up without statistical significance. Compared to the preoperative results, both groups reached higher results in all subscales of the score **(**Table 2**)**.

### Patient related outcome measurements results

The postoperative overall WOMAC scores showed no clinically relevant differences between the 2 study groups (*p* = 0.386) **(**Table 2**)** with a statistically significant difference of 6 points.

The pre- and postoperative level of pain after the VAS showed a significant decrease of pain during the follow-up period for both groups, but presented no significant differences between the 2 study groups (*p* = 0.161 & *p* = 0.973) **(**Table 2**)**.

### Complications

During the follow-up period no complications that required implant exchange, such as polyethylene wear, insert dislocation, aseptic loosening, or infection was observed.

## Discussion

The most important findings of the present study were that patients treated either with FB or MB insert reached similar postoperative results. However, MBs achieved superior results regarding ROM, which is in line with the existing literature [5, 17, 18, 25]. Functional and patient reported outcomes with satisfying survival rates are reported in published studies for FB and MB inserts in cemented TKA [19, 20]. The results of this present study support the operation method as a well-established standard and findings either for fixed and mobile bearings.

Differences in ROM between FB and MB inserts are interpreted as a variation of normal postoperative range of knee motion. Therefore, the partly superior results for MB insert in the present study have to be seen under a certain reservation. A prospective randomized study of Aglietti et al. also presented better ROM results for MB in the short term [26]. Nevertheless, a systematic literature review with a high amount of studies and patients included, presented no differences regarding postoperative range of motion [25]. Nether short- nor long-term follow-ups presented an advantage or disadvantage for one the insert types [5, 7, 27, 28].

Patient reported outcome scores showed satisfying postoperative results for both groups and presented significant better results for the FB group compared during the follow up period. Prospective randomized trials published in the literature could do not present this level of significance, but present similar postoperative WOMAC results [3, 29]. Despite statistically significant advantages for the FB group regarding the WOMAC score, the clinical relevance has to been seen controversial. The postoperative overall results in both study groups are satisfying compared to published literature and a translation to clinically relevance is difficult [26, 30].

The findings of this study present contradictory results between measured functional results and PROM results. A study by Shi et al. also mentioned, that the objective measured ROM does not appear to directly relate to the subjective experienced quality of movement [31]. It can be assumed that FBs may result in as more stable in patients’ subjective perceptions and may therefore present better PROM scores.

A study by Garling et al. reported, that the rotational effect of MB inserts is overestimated [32]. A fluoroscopy during movements showed that a limited rotation presumable due to impingement [33, 34]. These findings may limit the advantages of MB inserts and lead to non-significant results compared to FB platforms. Despite that there is no advise for the use of MB of FB inserts in the literature, is has to be mentioned that the comparability is limited due to the different implant types and their technical specifications that were used.

## Limitation

First, the sample size and the loss of follow-up limits this present study. Second, the applied scores such as KSS and WOMAC might not be sensitive enough to detect small differences between both groups. In addition, we did not use the forgotten joint score, which might have been superior with respect to discrimination of our results. Third, a longer follow-up period could have generated more knowledge regarding wear, aseptic loosening, and finally insert dislocation. Fourth, the absence of randomization limits the validity of the study. Fifth, our study only included cemented designs and it is unclear, whether our findings are comparable for cementless systems, which are more often used in mobile bearing TKA. However, we want to underline the benefit, that this is the first study in literature evaluating this topic with an adequate a priori power analysis.

## Conclusion

According to the findings, both inserts showed overall promising postoperative results, in terms of objective as well as subjective parameters, without clinically relevant significant differences between mobile and fixed inserts, except for ROM, which was superior in the mobile bearing group.

## Supplementary Information

**Additional file 1.**

## Data Availability

To enable full transparency, the anonymized raw data is uploaded as a additional file.
